# Cytotoxicity and Toxicity Evaluation of Xanthone Crude Extract on Hypoxic Human Hepatocellular Carcinoma and Zebrafish (*Danio rerio*) Embryos

**DOI:** 10.3390/toxics6040060

**Published:** 2018-10-09

**Authors:** Shazrul Fazry, Muhammad Akram Mohd Noordin, Salahuddin Sanusi, Mahanem Mat Noor, Wan Mohd Aizat, Azwan Mat Lazim, Herryawan Ryadi Eziwar Dyari, Nur Hidayah Jamar, Juwairiah Remali, Babul Airianah Othman, Douglas Law, Nik Marzuki Sidik, Yew Hoong Cheah, Yi Chieh Lim

**Affiliations:** 1 Faculty Science and Technology, Universiti Kebangsaan Malaysia, Bangi 43600, Selangor, Malaysia; akram_noordin@yahoo.com (M.A.M.N.); mahanem@ukm.edu.my (M.M.N.); azwanlazim@ukm.edu.my (A.M.L.); herry@ukm.edu.my (H.R.E.D.); njamar@ukm.edu.my (N.H.J.); juwairiah.remali@gmail.com (J.R.); airianah@ukm.edu.my (B.A.O.); 2 Tasik Chini Research Centre, Faculty Science and Technology, Universiti Kebangsaan Malaysia, Bangi 43600, Selangor, Malaysia; 3 Institute of Systems Biology (INBIOSIS), Universiti Kebangsaan Malaysia, Bangi 43600, Selangor, Malaysia; saladsanusi@gmail.com (S.S.); wma@ukm.edu.my (W.M.A.); 4 Faculty of Agro Based Industry, Universiti Malaysia Kelantan, Jeli Campus, Locked Bag 100, Jeli 17600, Kelantan, Malaysia; douglas.law@gmail.com (D.L.); nikmarzuki@umk.edu.my (N.M.S.); 5 ZACH Biotech Depot Private Limited, Cheras 43300, Selangor, Malaysia; yhcheah@zachbiotech.com; 6 Danish Cancer Society Research Centre, Strandboulevarden 49, 2100 Copenhagen, Denmark; yilim@cancer.dk

**Keywords:** xanthone, α-mangostin, HPLC, MTT proliferation assay, fish embryo toxicity test

## Abstract

Xanthone is an organic compound mostly found in mangosteen pericarp and widely known for its anti-proliferating effect on cancer cells. In this study, we evaluated the effects of xanthone crude extract (XCE) and α-mangostin (α-MG) on normoxic and hypoxic human hepatocellular carcinoma (HepG2) cells and their toxicity towards zebrafish embryos. XCE was isolated using a mixture of acetone and water (80:20) and verified via high performance liquid chromatography (HPLC). Both XCE and α-MG showed higher anti-proliferation effects on normoxic HepG2 cells compared to the control drug, 5-fluorouracil (IC_50_ = 50.23 ± 1.38, 8.39 ± 0.14, and 143.75 ± 15.31 μg/mL, respectively). In hypoxic conditions, HepG2 cells were two times less sensitive towards XCE compared to normoxic HepG2 cells (IC_50_ = 109.38 ± 1.80 μg/mL) and three times less sensitive when treated with >500 μg/mL 5-fluorouracil (5-FU). A similar trend was seen with the α-MG treatment on hypoxic HepG2 cells (IC_50_ = 10.11 ± 0.05 μg/mL) compared to normoxic HepG2 cells. However, at a concentration of 12.5 μg/mL, the α-MG treatment caused tail-bend deformities in surviving zebrafish embryos, while no malformation was observed when embryos were exposed to XCE and 5-FU treatments. Our study suggests that both XCE and α-MG are capable of inhibiting HepG2 cell proliferation during normoxic and hypoxic conditions, more effectively than 5-FU. However, XCE is the preferred option as no malformation was observed in surviving zebrafish embryos and it is more cost efficient than α-MG.

## 1. Introduction

Primary liver cancer is the second leading cause of cancer-related death worldwide [1]. In Malaysia, liver cancer is most common among people of Chinese ethnic descent, followed by Malays and Indians, the primary cause being a hepatitis B viral infection [2]. Despite the existence of many treatment options, such as curative surgical resection, chemotherapy, liver transplantation, radiofrequency ablation, and non-curative trans-arterial chemoembolization, the survival rate of patients is very low due to late diagnosis [3]. Doxorubicin, 5-fluorouracil (5-FU), and cisplatin are some of the commercial drugs used with systemic chemotherapy for liver cancer [4,5,6]. However, they can cause numerous side effects as normal healthy cells that are actively dividing, such as those in bone marrow, the intestinal lining, and hair follicles, are also affected. Another problem with chemotherapy for liver cancer is hypoxia-induced chemoresistance [7]. It has been reported that the action of 5-FU on cancer cells is reduced under hypoxic conditions (lack of oxygen in the microenvironment surrounding cancer cells) [8,9].

Xanthones are a group of organic compounds mainly found as secondary metabolites in higher plants and microorganisms. Natural xanthone derivatives are known to possess anti-cancer effects, which can be enhanced through the modification of substituents on the ring structure and their positions [10]. Previously reported pharmacologic properties of xanthone crude extract (XCE) include anti-cancer [11], antibacteria [12], and antidiabetic [13] effects. Interestingly, xanthone extracts have also shown protective properties against oxidative stress and inflammation in several organs, including the skin [14], bowel [15], cardiovascular system [16], and liver [17]. 

One of the XCE constituents, α-mangostin (α-MG), has been reported to promote cell cycle arrest and apoptosis in liver, colon, and prostate cancers [18,19,20]. More recently, the ability of xanthone derivatives to inhibit cancer progression in hypoxic conditions has also been discovered [21]. Despite its great potential, there is a lack of research evaluating xanthone efficacy and toxicity in-vivo. In this study, we evaluate the anti-proliferation effect of α-MG and XCE on human hepatocellular carcinoma (HepG2) cell lines compared to 5-FU and investigate to toxicity of all three compounds using zebrafish embryos. Zebrafish (*Danio rerio*) has emerged as a robust vertebrate model for drug discovery, especially in cancer research. Over the last two decades, zebrafish has been utilized in several areas of cancer research, including angiogenesis, metastasis, anti-tumor drug screening, and drug toxicity evaluation [22,23,24,25,26]. Hepatocellular carcinoma cell lines from zebrafish have been found to express similar genes to the human cell line, indicating their potential as a suitable animal model [27].

## 2. Materials and Methods

### 2.1. Xanthone Extract

XCE was extracted as previously described by Walker [28]. Briefly, 0.1 g of dried mangosteen pericarp was dissolved in a solvent mixture of 40 mL acetone/water (80:20) to effectively extract the XCE. The mixture was then placed in a 50 mL volumetric flask and left in a wrist-shaker for 30 min to ensure thorough mixing. The sample was filtered (0.45 μm) and dried using a freeze dryer (model no. 74200-30) (Labconco, Kansas City, MO, USA) for 150 min at −60 °C.

### 2.2. High Performance Liquid Chromatography (HPLC) Profiling of XCE 

High performance liquid chromatographic (HPLC) studies were conducted according to the procedures performed by Walker [28] at the Research and Instrumentation Center (CRIM), Universiti Kebangsaan Malaysia (UKM). An HPLC system Waters 2695 fitter with a UV spectrum and a Millenium32 Software Ver. 3.05.01 (GenTech Scientific, Arcade, NY, USA) was used. The subsequent HPLC analysis of XCE was carried out using a C18 analytical column (3.9 × 150 mm) and approximately 60-min gradient of 65–90% methanol in 0.1% formic acid with UV detection at 200–400 nm [29]. A sample injection volume was adjusted to 10 μL and introduced into the HPLC system at a flow rate of 1.0 mL/min for 1 h with pure α-mangostin (α-MG) used as the standard. The concentration of total α-MG in XCE was calculated by applying the peak area to the linear regression equations of pure α-MG calibration curves at concentrations ranging from 1–200 μg/mL (*n* = 3). The α-MG was purchased from Aktin Chemicals (Hi-tech Zone, Chengdu, China).

### 2.3. Cytotoxicity Assay

Human hepatocellular carcinoma (HepG2) and mouse skeletal muscle (L6) cell lines were cultured in Dulbecco’s Modified Eagle Media (DMEM) with a mixture of 15% Fetal Bovine Serum (FBS), 100 U/mL penicillin, and 100 μg/mL streptomycin. Cells were incubated under 5% carbon dioxide at 37 °C [30]. Cobalt chloride (100 μM) was used to induce cell hypoxia [31]. Cytotoxicity of XCE and α-MG were determined via MTT assay as described by Muniandy et al. [32]. As a drug control treatment, 5-FU was used. Treated cells were incubated for 48 h to determine the cytotoxic effect of XCE, α-MG, and 5-FU.

### 2.4. Zebrafish Embryos

For each independent experiment, zebrafish embryos from the same spawn of eggs were used, supplied by Biochemistry Department, Faculty of Biotechnology and Biomolecule Science (UPM). Zebrafish eggs were collected and chosen under light microscope 1 h post fertilization (hpf). These fertilized eggs were cleaned using distilled water and incubated at 28 °C in E3 medium (5 mM NaCl, 0.17 mM KCl, 0.33 mM CaCl_2_, 0.33 mM MgSO_4_, and 0.1% (*w*/*v*) methylene blue).

### 2.5. Zebrafish Embryonic Toxicity Test

The successfully fertilized zebrafish embryos (1 hpf) were exposed to various concentrations of XCE (7.81, 15.63, 31.25, 62.5, 125, 250 μg/mL), α-MG (0.78, 1.56, 3.13, 6.25, 12.5, 25, 50 μg/mL), and 5-FU (7.81, 15.63, 31.25, 62.5, 125, 250, 500 μg/mL) for 72 h in a 96-well plate. DMSO (1%) (Sigma-Aldrich, St. Louis, MO, USA) was used as a mock control. Each experiment was carried out with five technical repeats and repeated thrice. The survivability and condition of the embryos was captured under a light microscope (Olympus SZX10, Shinjuku-ku, TYO, Japan) using 40X and 100X magnifications. Animal care and all experimentation were conducted in compliance with the Organization for Economic Cooperation and Development (OECD) [33,34] and was approved by the UKM ethics committee (Animal ethic approval number: FST/2015/SHAZRUL/25-MAR./672-MAR.-2015-DEC.-2017) on 25 March 2015.

## 3. Results and Discussion

### 3.1. HPLC Profile of Xanthone Crude Extract

**Figure 1:**
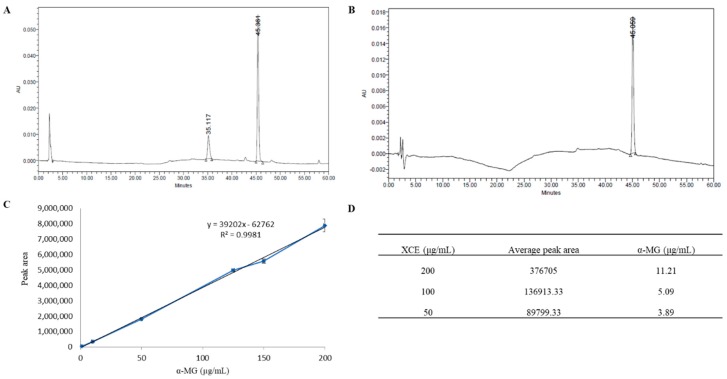
Crude xanthone extraction. High performance liquid chromatography (HPLC) chromatogram profile of (**A**) xanthone crude extract (XCE) and (**B**) pure α-mangostin (α-MG) samples. The same characteristic peak was identified at the same retention time of 45 min. Standard curve of pure α-MG (μg/mL) (**C**) was plotted to determine xanthone concentration in XCE. (**D**) showed the total α-MG content within XCE (μg/mL). Error bars showed the standard error of the mean (SEM).

HPLC analysis was used to identify the presence of α-MG in XCE obtained from mangosteen pericarp with pure α-MG used as the standard. XCE showed the characteristic peak of α-MG at the same retention time of 45 min as that of the standard, verifying the presence of α-MG in XCE (Figure 1A,B). Based on the standard curve of pure α-MG (Figure 1C), the concentration of α-MG was found to be 3.89, 5.09, and 11.2 μg/mL in 50, 100, and 200 μg/mL of XCE, respectively (Figure 1D). All concentrations of XCE used in this study were standardized using the content of α-MG within the XCE, assuming the ratio concentration of other compounds are relatively constant to the concentration of α-MG. The same strategy was employed to ensure repeatability of the experiment.

**Figure 2:**
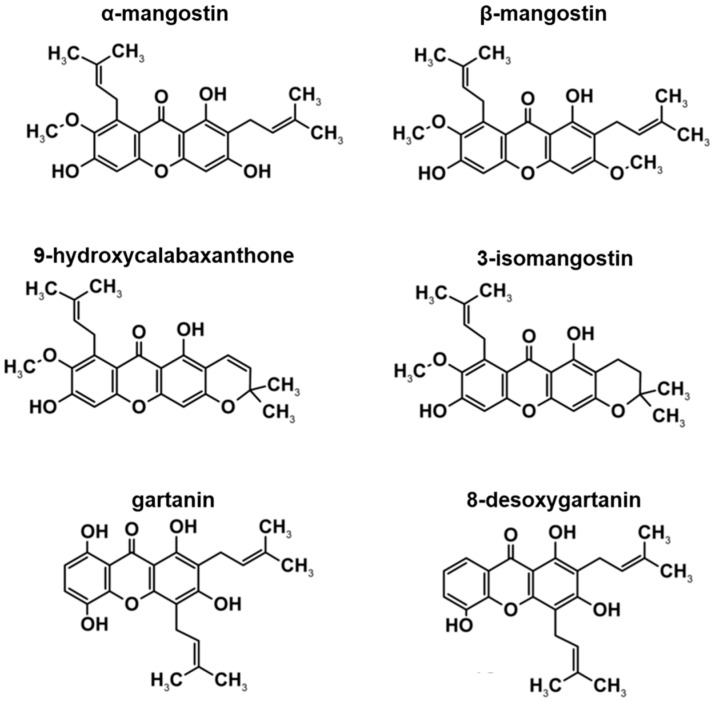
Chemical structures of α-mangostin (α-MG), β-mangostin (β-MG), 9-hydroxycalabaxanthone, 3-isomangostin, gartanin, and 8-desoxygartanin, which are reportedly present in XCE [21]. Figure is adapted from Walker [28] with permission from Elsevier (Copyright 2007).

The HPLC profile in this study, supports previous findings by Walker [28] as to the constituents of XCE. Previous studies have reported the discovery of xanthone derivatives, including α-MG, β-mangostin (β-MG), 9-hydroxycalabaxanthone, 3-isomangostin, gartanin, and 8-desoxygartanin (Figure 2). With the exception of α-MG, the concentration of other xanthone derivatives in XCE were not determined in this study. Many of the known functions of XCE have been widely studied and reviewed. These include: anticancer effects (including anti-proliferative, anti-carcinogenic and pro-apoptotic), antioxidant, anti-inflammatory, antibacterial, antimalarial, anti-obesity, neuroprotective, hepatoprotective, and cardioprotective abilities. However, most of these studies focus on the efficacy and function of α-MG, β-MG, and gartanin, in-vitro and in-vivo [35,36,37,38,39]. For example, α-MG was discovered when trying to increase apoptotic activity by activating caspase-3 and -9 activity and enhancing the MAPK/ERK pathway [36]. The derivative β-MG has been shown to inhibit the growth of breast cancer cell line MCF7, inducing apoptosis and halting the G2/M checkpoint in the p53-dependent pathway [37]. Similarly, gartanin was shown to induce apoptosis and stop cancer cell growth in-vivo by affecting the mTOR pathway [38]. How these three xanthone constituents function alone is very complex and may potentially make the XCE a more potent and effective anti-cancer substance than using pure α-MG alone. If existent, the antagonistic effect between all three constituents remains to be elucidated. To date, the specific functions of 9-hydroxycalabaxanthone, 3-isomangostin, and 8-desoxygartanin are yet to be fully characterized and determined.

### 3.2. Cytotoxicity of XCE and α-MG

**Figure 3:**
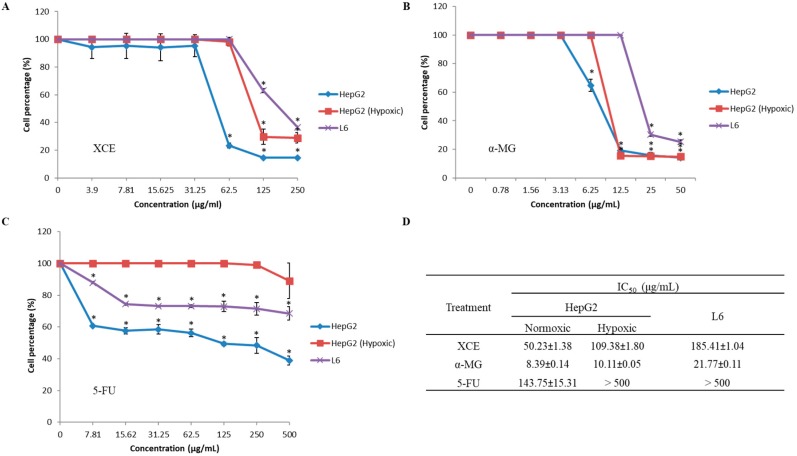
The cytotoxic effect of (**A**) XCE, (**B**) α-MG, and (**C**) 5-FU on HepG2 (both hypoxic and normoxic conditions) and L6 cell lines. (**D**) Cells were treated with a series of concentrations to obtain half maximal inhibitory concentration (IC_50_) of the treatment compound against cell survivability. All experiments were repeated thrice with five technical replicates. Error bars showed the SEM. Asterisk (*) showed the significant (*p* < 0.05) difference between cell groups at each concentration. Experiments were analyzed using single ANOVA.

The cytotoxic effects of XCE, α-MG, and 5-FU on HepG2 (both hypoxic and normoxic conditions) and L6 cell lines were assessed using an MTT assay. Figure 3 shows the percentage of surviving cells after treatment with a series of compounds or extract concentrations. The concentration of XCE, α-MG, and 5-FU needed to inhibit cell proliferation by 50% was determined by extrapolating values (IC_50_) from the graph. XCE (IC_50_ = 50.23 ± 1.38 μg/mL) and α-MG (IC_50_ = 8.39 ± 0.14 μg/mL) have higher cytotoxic effects on normoxic HepG2 cells compared to 5-FU (IC_50_ = 143.75 ± 15.31 μg/mL). While 5-FU did not inhibit normal L6 cell lines (IC_50_ > 500 μg/mL), both XCE and α-MG were shown to inhibit L6 cells, albeit at a lesser degree, compared to normoxic HepG2 cells (IC_50_ = 185.41 ± 1.04 μg/mL and 21.77 ± 0.11 μg/mL, respectively). This is an interesting discovery, as reports on the effect of mangosteen extracts in general and α-MG in particular, on normal cell lines have not been widely reported [40]. We hypothesize that the reason XCE is less cytotoxic, compared to α-MG, may be due to other bioactive compounds in the extract, which antagonize the effect of α-MG. Finally, the efficacy of both XCE and α-MG on hypoxic HepG2 cells (IC_50_ = 109.38 μg/mL and 10.11 ± 0.05 μg/mL, respectively) was also determined. Consistent with previous findings, 5-FU did not appear to exhibit any cytotoxic effect on hypoxic HepG2 cells [8,9].

**Figure 4:**
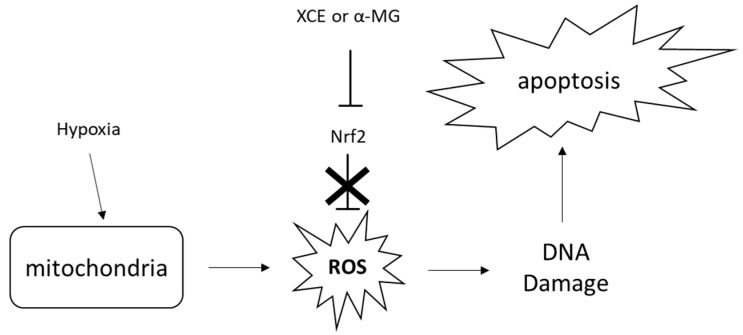
During hypoxia, mitochondrial reactive oxygen species (ROS) are induced exponentially. This could lead to DNA damage, which could induce apoptosis. Nrf2 is usually activated during this event to trigger an event that neutralizes ROS. Inhibition of NRF2 by XCE or α-MG disrupts ROS neutralization, promoting cell death by apoptosis.

A popular drug used to treat cancer, 5-FU [4] can inhibit cell division by inhibiting DNA replication at the DNA replication fork [41]. However, this strategy may not be as effective as expected, as cells are equipped with machinery to bypass or repair this blockade. Furthermore, hypoxic cancer cells may not be affected by 5-FU, as they may not divide at the same rate as normoxic cancer cells, thereby limiting the incorporation of 5-FU to their DNA. Both XCE and α-MG have been reported to inhibit nuclear factor erythroid 2–related factor 2 (Nrf2) [42], which triggers the antioxidant response to detoxify cells from intracellular reactive oxygen species (ROS) [43,44]. During hypoxia, ROS increase due to the further reduction state of the electron transport chain within mitochondria [45]. With Nrf2 inhibited by XCE or α-MG, cells might be overwhelmed with ROS, which could damage the cells’ DNA. This in turn, may induce apoptosis within the cell [43], which could explain why apoptosis is seen in cells treated with either XCE or α-MG [35] (Figure 4).

### 3.3. Toxicity of XCE and α-MG on Zebrafish Embryos

**Figure 5:**
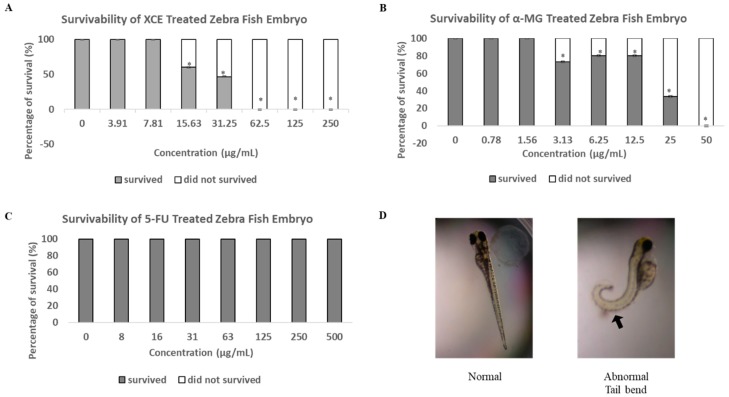
The toxicity of (**A**) XCE, (**B**) α-MG, and (**C**) 5-FU on zebrafish embryos. Experiments were statistically analyzed using two-tailed Student’s t-test. Asterisk (*) shows the significant (*p* < 0.05) difference between the treatment group and the untreated group (the concentration of 0 μg/mL of the compound or extract). All experiments were repeated five times. (**D**) shows the representative image of tail-bend malformation of embryos (arrow) treated with α-MG. No XCE and 5-FU treated embryos were found to exhibit any malformation.

While the cytotoxic effect of α-MG and xanthones from mangosteen has been established and supported, the safety of these compound in vivo, especially on developing embryos has not been elucidated. In this study, we showed that both XCE and α-MG caused embryonic mortality at 15.63 μg/mL and 3.13 μg/mL, respectively (Figure 5). Furthermore, a mortality rate of 100% was observed at a concentration of 62.5 μg/mL for XCE and 50 μg/mL for α-MG and onwards. The typical endpoint of embryonic development in zebrafish is when no clumps form in the embryo, a heartbeat is present, body segments are completely formed, and the tail is completely separated from the yolk [46,47,48]. Interestingly, our data showed that all surviving α-MG treated embryos at 12.5 μg/mL and above exhibited a tail bend malformation, indicating α-MG may inhibit some developmental gene expression (Figure 5D). Presence of other malformations in α-MG treated embryos was not observed. XCE and 5-FU treated embryos did not exhibit any mortality or malformation (Figure 5C). As discussed previously, the lack of malformation in XCE treated embryos may be due to the existence of other compounds within XCE that may antagonize the side effects of α-MG.

We hypothesize that the Cysteine-rich motor neuron 1 (CRIM1) protein associated pathway may be directly or indirectly affected by α-MG. CRIM1 is a bone morphogenetic protein (BMP) antagonist, which regulates embryogenesis (during neurulation and hemangiogenesis) [49,50]. During the development of chicken and mice, CRIM1 can be observed localizing in the nerve tube area, assisting backbone and neural tube formation [51]. A report has shown that disruption of the *crim1* gene can cause a tail bend malformation in zebrafish embryos [52], supporting this hypothesis further. Interestingly, studies have also shown that the knockdown of CRIM1 suppresses the ability for lung cancer cells to migrate and adhere properly [53,54], which could explain why the HepG2 cells in our study were not able to propagate properly once treated with α-MG. Nevertheless, further investigations are required to determine the relationship between the CRIM1 associated pathway and α-MG.
